# Stress-Controlled Creep–Fatigue of an Advanced Austenitic Stainless Steel at Elevated Temperatures

**DOI:** 10.3390/ma15113984

**Published:** 2022-06-03

**Authors:** Zeinab Y. Alsmadi, Hamdy Abouelella, Abdullah S. Alomari, K. L. Murty

**Affiliations:** 1 Department of Nuclear Engineering, North Carolina State University, Raleigh, NC 27695-7909, USA; zalsmad@ncsu.edu (Z.Y.A.); hsmostaf@ncsu.edu (H.A.); murty@ncsu.edu (K.L.M.); 2 Nuclear Science Research Institute, King Abdulaziz City for Science and Technology (KACST), P.O. Box 6086, Riyadh 11442, Saudi Arabia

**Keywords:** creep–fatigue, Alloy 709, stress-controlled, softening, ratcheting, hysteresis loops, high-temperature deformation

## Abstract

Creep–fatigue interaction occurs in many structural components of high-temperature systems operating under cyclic and steady-state service conditions, such as in nuclear power plants, aerospace, naval, and other industrial applications. Thus, understanding micromechanisms governing high-temperature creep–fatigue behavior is essential for safety and design considerations. In this work, stress-controlled creep–fatigue tests of advanced austenitic stainless steel (Alloy 709) were performed at a 400 MPa stress range and 750 °C with tensile hold times of 0, 60, 600, 1800, and 3600 s, followed by microstructural examinations. The creep–fatigue lifetime of the Alloy 709 was found to decrease with increasing hold time until reaching a saturation level where the number of cycles to failure did not exhibit a significant decrease. Softening behavior was observed at the beginning of the test, possibly due to the recovery of entangled dislocations and de-twining. In addition, hysteresis loops showed ratcheting behavior, although the mean stress was zero during creep–fatigue cycling, which was attributed to activity of partial dislocations. Microstructural examination of the fracture surfaces showed that fatigue failure dominated at small hold times where the cracks initiated at the surface of the sample. Larger creep cracks were found for longer hold times with a lower probability of dimpled cavities, indicating the dominance of creep deformation. The results were compared with other commonly used stainless steels, and plausible reasons for the observed responses were described.

## 1. Introduction

One of the advantages of nuclear power plants is the ability to operate at higher temperatures, leading to increased thermal efficiency; however, thermal transients at startups and shutdowns lead to large deformation in the structural materials due to thermal expansion and contraction causing fatigue damage [1,2,3]. In addition, long steady-state operation periods at higher temperatures and stresses can lead to creep damage [1]. The combined effects of creep and fatigue damages result in reduced lifetimes due to the damage caused by reversed loading at high temperatures, combining effects of both fatigue and creep [4]. Creep–fatigue interaction should be considered in the design because both fatigue and creep exhibit different microstructural mechanisms; thus, extensive studies have been conducted to investigate the effect of combined creep–fatigue loading conditions [5,6,7]. Most of the creep–fatigue studies focused on strain-controlled creep–fatigue mimicking operating conditions [4,5,8,9]. The lower- and upper-end fittings of the fuel bundle and many other parts of the reactor core are not allowed to strain freely; thus, the creep–fatigue responses under stress-controlled test conditions are closely related to potential damage to such parts instead of strain-controlled conditions. There are many differences between strain- and stress-controlled creep–fatigue tests. In strain-controlled tests, the material exhibits stress relaxation during hold times while the strain is maintained constant throughout the hold time. Therefore, the stress required to sustain constant strain decreases with time. This reflects an increase in the permanent plastic deformation, along with decreased elastic deformation. In stress-controlled creep–fatigue tests, however, the stress is kept constant during hold time so that creep occurs. If the hold time is long enough, a steady-state regime appears after the primary creep region. Analyses of stress relaxation during hold time of a strain-controlled creep–fatigue test contain a fitting of relaxation functions that might be a source of error propagation, depending on the quality of fitting parameters [4,5,8,9,10]. Creep deformation during the hold time in stress-controlled creep–fatigue tests is straightforward to analyze and more accurate. In strain-controlled creep–fatigue tests, the strain is the independent variable, and the stress is the dependent variable. In other words, a material’s response to the applied strain is reflected in the change of stress required to reach preset strain conditions. In stress-controlled creep-fatigue tests, by contrast, the stress is the independent variable, and the strain is the dependent variable, which means the change in strain versus time reflects the deformation happening inside the material due to the applied stress. Furthermore, in stress-controlled creep–fatigue tests with non-zero mean stress, cycles may not be fully reversed. This results in progressive strain accumulation [1,6,11], often called “ratcheting strain”, which is defined as the progressive directional strain causing a shift in the hysteresis loop along the strain axis. Ratcheting is one of the major phenomena that can affect the life of engineering components and must be considered in the design and life evaluation of materials operating at high temperatures. Generally, ratcheting strain is accumulated in the mean stress direction (i.e., positive ratcheting strain in the presence of tensile mean stress and negative ratcheting strain in the presence of compressive mean stress), while no ratcheting effect is expected when tests are performed under zero mean stress [11].

Due to their excellent mechanical properties at elevated temperatures and sufficient corrosion/oxidation resistance, austenitic stainless steels have been widely used in both conventional and advanced reactor technologies [12]. One of the newly developed advanced austenitic stainless steels is Alloy 709 (belongs to the family of Fe-25Ni-20Cr (wt. %) stainless steels), which is a strong candidate structural material for next-generation nuclear reactors [13,14]. Preliminary investigations have shown that Alloy 709 is compatible with a sodium-rich environment and thermally stable with good high-temperature strength due to the existence of niobium (Nb) [15,16]. However, understanding micromechanisms governing high-temperature creep–fatigue behavior of this alloy is not well-established. Few authors have carried out creep–fatigue tests in order to understand the response of Alloy 709 at elevated temperatures [10,14,17,18,19]. Alsmadi et al. [10,14,19] have performed high-temperature creep–fatigue tests in strain-controlled mode under different experimental conditions to study the effect of tensile hold time (0, 60, 600, 1800, and 3600 s), temperature (650 and 750 °C), and strain range (0.6–1.2%) on the creep–fatigue life of Alloy 709. The creep–fatigue life of the Alloy 709 was shown to decrease with increasing tensile hold time, temperature, and strain range. The microstructural characterization revealed transgranular fatigue cracks that developed into intergranular cracks and eventually resulted in premature failure of the alloy. Shaber et al. [18] investigated fatigue and creep–fatigue crack growth in Alloy 709 at high temperatures (550, 600, and 700 °C) at different hold times (0, 60, and 600 s), and they observed that crack growth rates were independent of hold times at 600 °C but increased slightly at 700 °C. Furthermore, creep–fatigue tests in strain-controlled mode with 30 min tensile hold times were conducted at 550 and 650 °C by Porter et al., who concluded that the less stress relaxation at lower temperatures was due to the differences in slip behavior, dynamic recovery, precipitate evolution, and dynamic strain-aging effects [17]. However, no single study, to the best of our knowledge, has been conducted on the creep–fatigue interaction in Alloy 709 in stress-controlled mode.

In this work, the creep–fatigue interaction of Alloy 709 was investigated under stress-controlled conditions at 750 °C and at a 400 MPa stress range with tensile hold times of 0, 60, 600, 1800, and 3600 s, at a stress rate of 80 MPa/s with a stress ratio of R = −1. The results were thoroughly discussed in terms of creep–fatigue life, along with the behavior of softening and the ratcheting strain and were compared with experimental data for other alloys.

## 2. Materials and Methods

### 2.1. Materials

**Figure 1:**
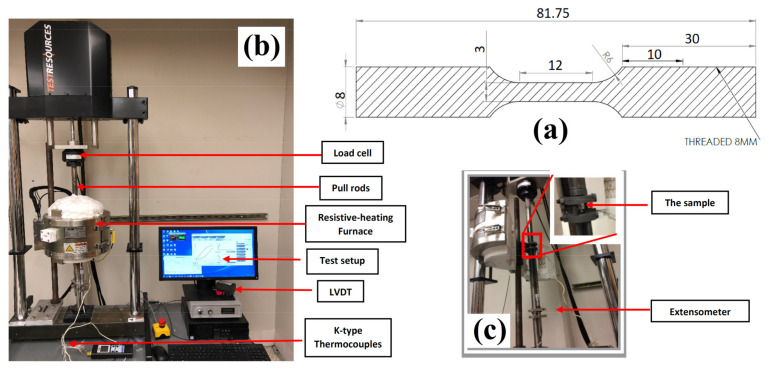
(**a**) Alloy 709 specimen geometry (dimensions in mm), (**b**) experimental electrodynamic testing machine setup for creep–fatigue tests, and (**c**) extensometer with LVDT.

The chemical composition of Alloy 709 investigated in this study is shown in Table 1. The as-received plate developed by ORNL had undergone hot rolling, followed by heat treatment at 1100 °C with subsequent quenching. Test specimens were machined from the as-received plate along the rolling direction with 3 mm gauge diameter and 12 mm gauge length, as per the drawing in Figure 1a.

### 2.2. Experimental Methods

**Figure 2:**
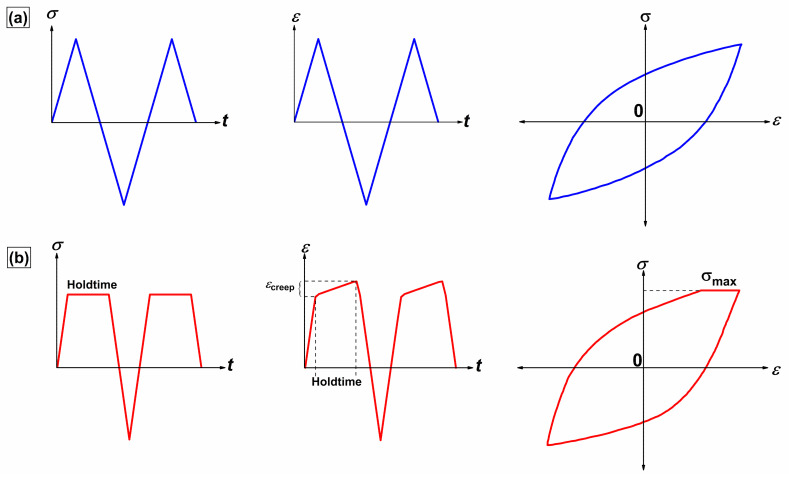
Schematics of loading cycle of stress-controlled creep–fatigue tests depicting stress–time, strain–time, and stress-strain curves (**a**) without a hold time and (**b**) with hold times imposed at tensile peak stress.

Stress-controlled creep–fatigue tests were performed in an electrodynamic creep–fatigue testing machine from Test Resources Company (Shakopee, MN, USA) (Figure 1b), following ASTM standard E2714-13. Displacements were monitored using a linear variable differential transducer (LVDT) attached to a high-temperature extensometer fixture. The electrodynamic creep–fatigue testing machine was equipped with a two-region furnace from Applied Test Systems (ATS, Butler, PA, USA), where the temperature was measured and controlled using two k-type thermocouples and furnace thermocouples (Figure 1c). The tests were conducted at a stress range of 400 MPa under fully reversed loading (R = −1) with a loading rate of 80 MPa/s and hold times of 0, 60, 600, 1800, and 3600 s. Loading started in the tensile regime, where the hold time was applied at the maximum tensile stress in every cycle. Figure 2 shows schematics of the loading cycle of the stress-controlled creep–fatigue tests in terms of stress–time, strain–time, and stress-strain curves with and without a hold time imposed at the tensile peak stress. Triangular waveforms appeared without a hold time, whereas the imposed hold time resulted in a trapezoidal waveform. Moreover, optical microscopy and scanning electron microscopy (SEM) were employed to examine the microstructures and fracture surfaces. Before performing microstructural characterization, the fractured samples were ultrasonically cleaned in acetone for 3 h before examining their morphology under SEM (ThermoFisher FEI Quanta 3D FEG) located at Advanced Instrumentation Facility (AIF) at NC State University, Raleigh, NC, USA The sample preparation for optical microscopy involved first cutting through the transverse direction within the gauge length using a low-speed saw. The cut specimen was then mounted in a cold-setting epoxy and polished using grinding wheel embedded with different grades of sandpaper in the following order: 400, 600, 800, and 1200. The ground surface was then polished with diamond suspension to produce a smooth surface with mirror-like finish. Finally, the specimens were etched using a chemical solution consisting of water, hydrochloric acid, and nitric acid in a 1:1:1 ratio.

## 3. Results and Discussion

### 3.1. Creep–Fatigue Life and the Strain Behavior

**Figure 3:**
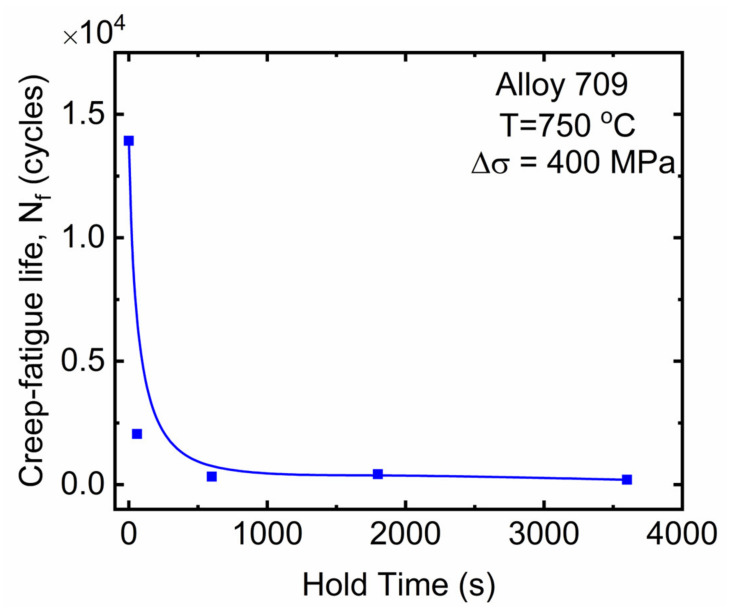
Creep–fatigue life of Alloy 709 at different hold times under stress-controlled condition at 750 °C.

Figure 3 shows the number of cycles to failure versus tensile hold time exhibiting decreased creep–fatigue life with increasing hold time. This result is consistent with those reported in various alloys including austenitic stainless steels [20], ferritic steels [21], and nickel-based super-alloy [22], where the introduction of a hold time anywhere in the loading cycle reduced the fatigue life compared to that of continuous cycling. Additionally, the creep–fatigue life significantly dropped from continuous cycling to the point where hold times were introduced, resulting in a decrease in the durability of the alloy, even at short hold times of 60 s, compared with its “pure” fatigue resistance. This significant drop was also observed in other materials such as nickel-based super-alloys, which was attributed to the higher transgranular crack propagation during hold times [8]. As the hold time further increased, the number of cycles to failure did not exhibit a significant decrease, indicating that longer hold times did not impose further damage. This saturation behavior has also been observed in Alloy 709 under strain-controlled conditions [10,14,19].

**Figure 4:**
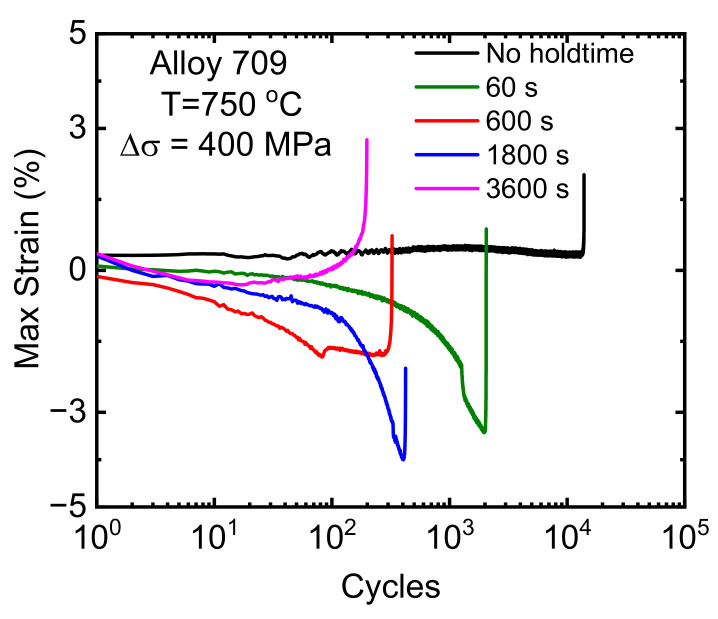
Maximum strain as a function of number of cycles for different hold times during stress-controlled creep–fatigue tests.

The change in the maximum strain at each hold time until failure versus the number of cycles is depicted in Figure 4. As shown, the plastic strain with no hold time (0 s) slowly increased at longer hold times, and the maximum strain evolved in the negative regime due to softening effect, as is explained below. However, at the 3600 s hold time, the maximum strain continued to rapidly increase at the end cycles (Figure 4). 

**Figure 5:**
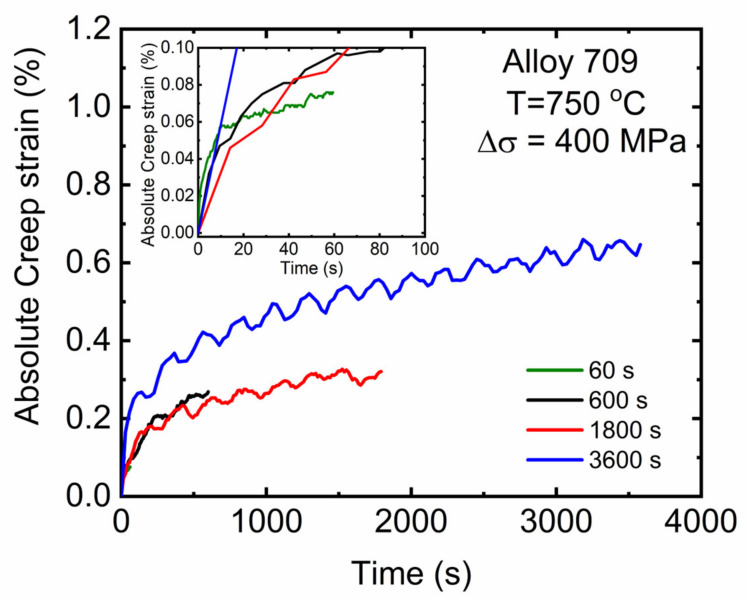
Creep strain accumulated during hold time corresponding to half-life cycle during stress-controlled creep–fatigue tests for different hold times.

For the time-dependent plastic strain (creep), Figure 5 shows the absolute value of the creep strain accumulated during the half-life cycle at different hold times. The creep strain mainly represented the primary creep regime where the strain rate at the beginning of hold duration decreased with increasing hold time. However, the creep curve at 3600 s hold time entered the steady-state regime with an evaluated steady-state strain rate of 6.1 × 10^−5^ s^−1^. The values of strain rates obtained at all examined hold times were found to be very close to those observed in creep tests where a value of minimum creep rate of 7.51 × 10^−7^ s^−1^ was observed at the same examined temperature (750 °C) and applied stress (200 MPa) [23].

### 3.2. Ratcheting Behavior

**Figure 6:**
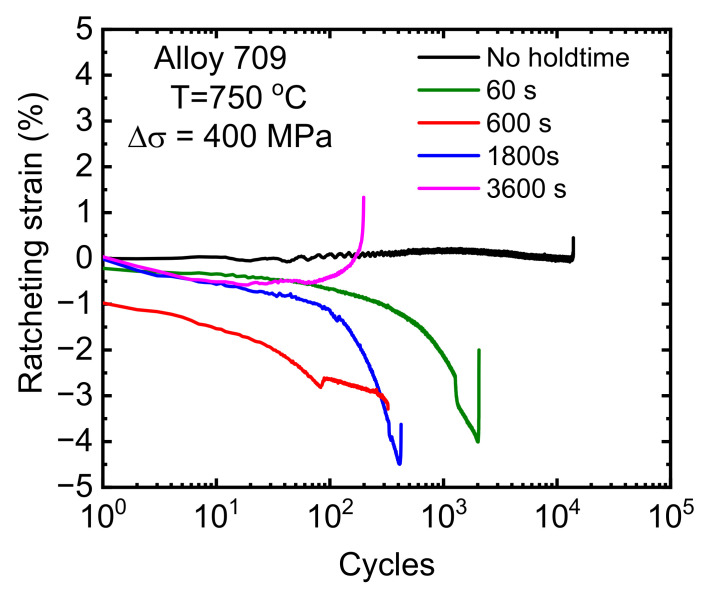
Ratcheting strain (defined as mean value of maximum and minimum strain in one cycle) versus number of cycles for different hold times during stress-controlled creep–fatigue tests.

**Figure 7:**
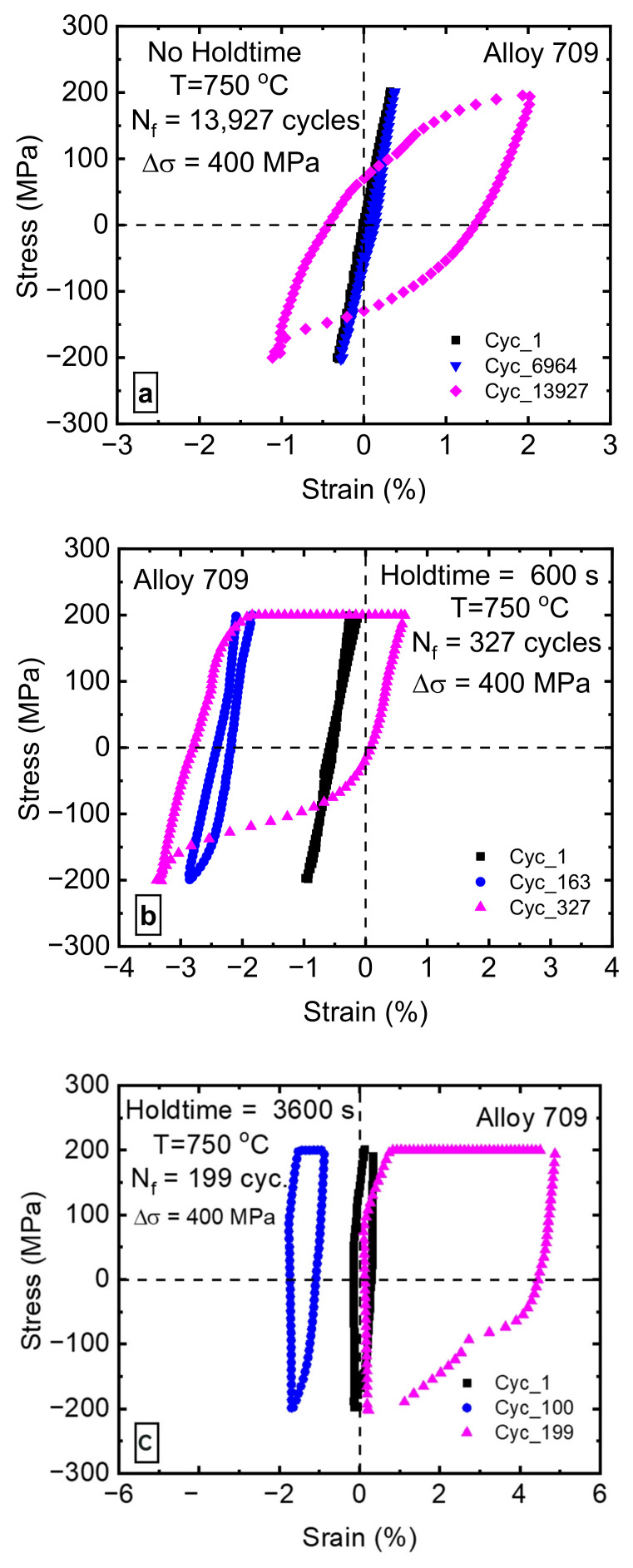
Hysteresis loops (first, midlife, and last cycles) of the stress-controlled creep–fatigue tests at 750 °C and hold times of (**a**) 0 s, (**b**) 600 s, and (**c**) 3600 s.

To examine the ratcheting behavior of Alloy 709, ratcheting strain (defined as the mean value of the maximum and minimum strain in one cycle) as a function of the cycle number is shown in Figure 6. Furthermore, hysteresis loops at the first, middle, and final cycles are plotted as a function of strain at different cycles and different hold times (Figure 7). When no hold time was introduced, as shown in Figure 7a, hysteresis loops exhibited plastic strain increase (i.e., the loop widened) with an increasing number of cycles. The total strain during tension and compression was found to be nearly fully reversed when no hold time was imposed. Theoretically, there should be no ratcheting strain at a stress ratio of Rn = −1 (i.e., zero mean stress) [24]; however, after introducing a hold time, a small amount of ratcheting strain accumulation was observed during stress-controlled, low-cycle creep–fatigue tests. This unusual ratcheting behavior became more pronounced as hold times were introduced at the peak tensile stress. Shifts in the hysteresis loops toward the negative and positive strain directions were observed, as shown in Figure 7b,c. Several studies have reported the occurrence of ratcheting during creep–fatigue [5,20,25,26,27,28]. In 2.25Cr1MoV steel, Zhao et al. observed ratcheting strains when holding periods were introduced at the peak and peak/valley stress waveforms [28]. Similar results have also been reported in a 9Cr–1Mo martensitic steel [5], 9–12%Cr steel [26], nickel-based super-alloy [8], 316L stainless steel [20], and 304 stainless steel [25]. Table 2 summarizes the creep–fatigue test parameters (stress amplitudes, mean stresses, and hold times) and proposed damage mechanisms for various materials from the literature where cyclic ratcheting has been observed. All these studies were performed at non-zero mean stresses, where the ratcheting strains were attributed to the presence of mean stress [11]. As the present study was conducted at zero mean stress, the ratcheting strain observed in Alloy 709 was distinct from that reported in the literature. To the best of our knowledge, there are no prior creep–fatigue studies on stainless steels where ratcheting strain was observed under zero mean stress. The ratcheting phenomenon occurs when the permanent strain accumulation is not fully reversed in cyclic loading due to, for example, a non-zero mean stress loading. The increase amount and the direction of the ratcheting strain are influenced by many factors, such as peak stress, mean stress, stress ratio, stress rate, and hold time [11]. When a hold time is imposed at the peak tensile stress under stress-controlled creep–fatigue test, two types of strain accumulation can occur: (1) time-independent plastic strain due to ratcheting behavior and (2) time-dependent plastic strain due to creep during hold time [11]. Further attempt to interpret such unconventional behavior based on softening is illustrated below.

### 3.3. Softening Behavior

Various studies have reported softening behavior in Alloy 709 when tested at 750 °C or beyond, where detailed microstructural analyses have been performed [7,9,14,23,29,30]. Zhao et al. studied the tensile behavior and microstructure evolution of Alloy 709 from room temperature to 900 °C using in situ x-ray diffraction and found that material hardening dominated up to 500 °C, where dynamic strain aging started to occur [29]. Softening was found to be the dominating behavior from 700 to 900 °C, attributed to dynamic recovery and recrystallization. Lall et al. have performed strain-controlled creep–fatigue tests at 750 °C and found intergranular cavitation at longer hold times [9,30]. They showed that twin boundaries were impeding dislocation motion at longer hold times. For a better understanding of ratcheting and the shift of the hysteresis loop to the strain regime, the applied stress is the independent variable, and the strain is the dependent variable in stress-controlled creep–fatigue tests. It means that mechanical behavior is identified by observing the change in the maximum strain range in each cycle.

**Figure 8:**
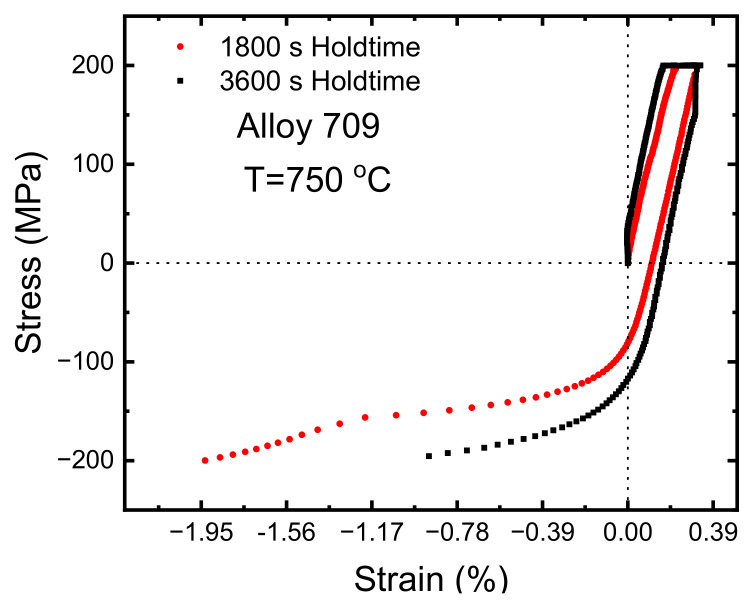
Loading cycle of the stress-controlled creep–fatigue tests depicting strain vs. stress for 1800 and 3600 s hold times.

One of softening manifestations is the sigmoidal hysteresis loops (Figure 7), rather than symmetric loops [31], where de-twining is most probably due to the activity of partial dislocations. Additionally, the unique orientation relationship between the parent grains and the twin grains favors de-twining during loading. Softening due to de-twining and increased mobile dislocations density causes decreases in the yield and ultimate tensile strengths of the specimen. To better understand of the sequence of events, Figure 8 shows the loading cycles for two different hold times. The test started with a tensile loading rate of 80 MPa/s and then the hold time was applied. At the end of the hold time, the load decreased to zero, at which point compressive stress was applied at the same rate. Due to softening, compressive stress applied after creep hold time was expected to cause more negative strains. This may be why compressive strain in the loading cycle was larger than that observed in the tension and holding periods (Figure 8). This caused the whole hysteresis loop of the next cycle to be totally in the compressive strain regime. Moreover, as the number of cycles increased, dislocations were consumed with time. The accumulation of plastic strain due to tensile creep led to ratcheting, even though the mean stress was zero, which forced the hysteresis loop to drift back to the positive strain regime. To understand the extent to which the hysteresis loop associated with each hold time shifts to the negative strain regime and drifts back to the positive strain, we recall that both dislocation recovery and consumption rate are time-dependent processes. In short, in hold time tests such as the 60 s hold time (Figure 7a), more than one cycle was needed to cause material softening and shift to the negative strain regime, as mentioned above. Wärner [31] studied the high-temperature fatigue of austenitic stainless steels, and found that dynamic recrystallization can occur if a certain critical strain is reached. The number of cycles needed to reach this critical strain depends on the dwell time applied in each test. Once this critical strain is reached, dynamic recrystallization starts to occur, leading to softening. Mobile dislocations liberated as a result of softening start to be annihilated, and the positive strain starts to accumulate in the tensile regime; however, the hold time is not long enough to enter steady-state creep regime and accumulate enough positive strain, thereby shifting the whole hysteresis back to the positive strain. Therefore, the final hysteresis loop is partially in the positive strain regime. For intermediate hold times such as 600 s, the hold time within the loading cycle was enough to accumulate critical strain leading to material softening. This could have caused the first cycle hysteresis to be in the negative strain regime (Figure 7b) with the portion of the hysteresis loop in the positive regime being larger than that for the 60 s hold time. For hold times such as 3600 s, the hold time was long enough to enter the steady-state creep regime during tensile loading (Figure 7c), which accumulated enough positive strain to shift the whole hysteresis back to the positive strain regime. During pure fatigue tests, i.e., zero hold time, the frequency of cyclic loading is too fast to allow twin boundary annihilation, leading to increase in the mobile dislocation density. However, at the end of the lifetime, softening was observed from the increased width of the hysteresis loops, so further investigations need to be carried out to address this complex phenomenon.

**Figure 9:**
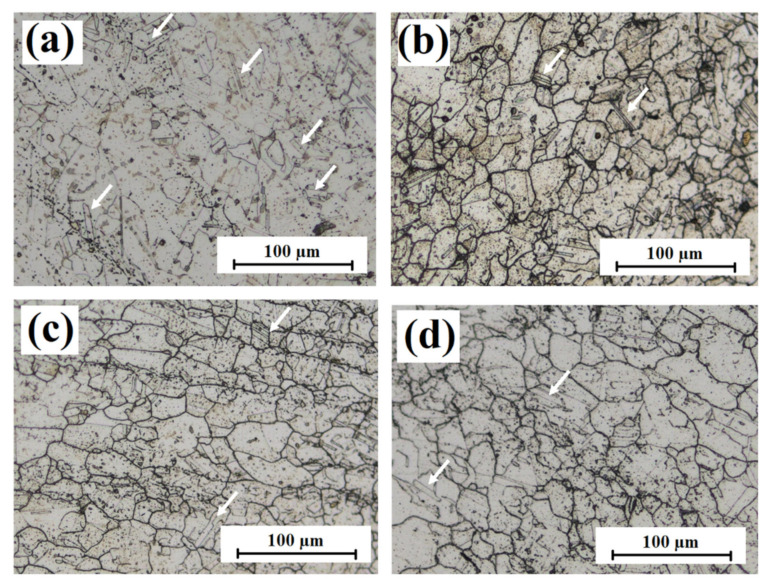
Confocal images of Alloy 709: as-received condition (**a**) and post-deformed under stress-controlled creep–fatigue tests for three different hold times: (**b**) 0, (**c**) 600, and (**d**) 3600 s. White arrows in the micrographs indicate twin boundaries.

Another manifestation of deformation by de-twining was illustrated in previous strain-controlled creep fatigue studies on Alloy 709, which revealed remarkable softening behavior. Higher dislocation density observed after testing ensured that piled up dislocations in virgin samples were recovered by stress-assisted processes. Unlike the low dislocation density observed in the as-received Alloy 709 [7], higher-density mobile dislocations developed after testing. Dislocations govern deformation behavior, implying a softer material with higher deformation tendency [1]. Creep–fatigue test initiated with tensile loading rate from 0 to 200 MPa in 2.5 s, followed by hold time, results in recovery of tangled dislocations leading to softening. Dislocations are recovered by climbing over obstacles in a stress-assisted diffusion process or by activity of partial dislocations leading to the annihilation of coherent/incoherent twin boundaries (de-twining). Figure 9 shows optical images of the as-received microstructure with a high density of twin boundaries compared with that following deformation (Figure 9b–d). When twin probability decreased, twin boundaries became more finely spaced in the tested samples along with shrinkage of twin planes. These observations were the manifestation of de-twining, as reported by many researchers in different metals [32,33,34,35].

**Figure 10:**
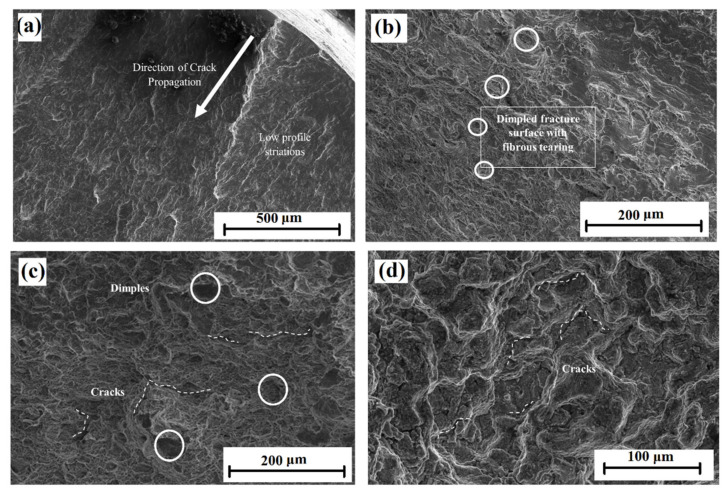
SEM fractographs of Alloy 709 deformed during stress-controlled creep–fatigue tests at 750 °C: without hold time (**a**,**b**), 600 s hold time (**c**), and 3600 s hold time (**d**).

The fracture surfaces of Alloy 709 subjected to stress-controlled creep–fatigue tests at different hold times were characterized using scanning electron microscopy (SEM), and Figure 10a,b depicts the fracture surface after the fatigue test with no hold time. Low profile striations are observed in Figure 10a, indicating fatigue failure along with the direction of the crack propagation initiated at the surface of the sample; Figure 10b shows the dimpled fracture surface with the fibrous tearing of the microstructure. At 600 s of hold time, the formation of short cracks was noted in the inner region of the cross-section (Figure 10c), and such cracks indicated creep crack formation along with dimpled ductile voids due to fatigue. At the 3600 s hold time, larger cracks were found (Figure 10d), indicating dominant creep deformation with a lower probability of dimpled cavities. This agrees with Figure 5, where creep during 3600 s hold time reached the steady-state regime, allowing more creep deformation to occur compared with all other tests.

## 4. Conclusions

The creep–fatigue interaction of Alloy 709 was investigated under stress-controlled conditions at 750 °C and 400 MPa stress range with tensile hold times of 0, 60, 600, 1800, and 3600 s at a 80 MPa/s stress rate. The following conclusions were drawn:

1. Creep–fatigue life was found to decrease with increasing hold time, where the introduction of a hold time anywhere in the loading cycle reduced the fatigue life compared with that of continuous cycling.
2. As the hold time further increased, the number of cycles to failure did not exhibit a significant decrease, indicating that longer hold times did not impose further damage.
3. The values of strain rates obtained at all examined hold times were found to be very close to those observed in creep tests, indicating the accumulation of creep damage during hold times.
4. Although the tests were performed at zero mean stress (stress ratio, R = −1), hysteresis loops exhibited ratcheting when hold time was introduced at the peak tensile stress where the accumulation of plastic strain was found toward both negative and positive strain directions. This anomalous behavior was interpreted in terms of softening behavior due to activity of partial dislocations leading to de-twining during the creep deformation in each cycle.
5. Softening behavior was observed at the beginning of the test due to the recovery of entangled dislocations and de-twining.
6. Microstructural examination of the fracture surfaces revealed that fatigue failure dominated at small hold times where the cracks initiated at the surface of the sample, while larger cracks were found with a lower probability of dimpled cavities due to creep at longer hold times.

Further investigations are underway to study the effect of mean stress on the ratcheting behavior of Alloy 709, as well as detailed microstructural studies using transmission electron microscopy (TEM).

## Figures and Tables

**Table 1:** Chemical composition (wt.%) of Alloy 709.

Element	Wt.%
Ni	24.9
Cr	19.93
Mo	1.51
Si	0.44
Nb	0.26
N	0.148
C	0.07
Ti	0.04
P	<0.014
B	0.0045
S	<0.001
Mn	0.91
Fe	Bal.

**Table 2:** Creep–fatigue test parameters (stress amplitudes, mean stresses, and hold times) and proposed damage mechanisms for various materials from the literature where cyclic ratcheting was observed.

Material	Stress Range (MPa)	Mean Stress (MPa)	Hold-Time (s)	Proposed Damage Mechanism	Ref.
Udimet 720 nickel-based superalloy	650–800	325–400	1–50 at peak stress	Inter-granular damage dominates	[8]
2.25Cr1MoV steel	525–600	100–30	2–60 at peak and peak/valley stress	Creep damage dominates at longer hold time (>5 s)	[28]
316L SS	385–535	118–26	1–5 at peak stress	Abrupt displacement jumps due to dynamic strain aging	[20]
304 SS	400–600	40	5–10 at peak stress	Creep damage dominates due to the viscosity of the material	[25]
Alloy 709	400	Zero	60–3600 at peak stress	Combined creep and fatigue damage	This study

## Data Availability

Not applicable.
